# Re-collection of *Dermeaprunus* in China, with a description of *D.chinensis* sp. nov.

**DOI:** 10.3897/mycokeys.50.32517

**Published:** 2019-04-04

**Authors:** Ning Jiang, Cheng-Ming Tian

**Affiliations:** 1 The Key Laboratory for Silviculture and Conservation of the Ministry of Education, Beijing Forestry University, Beijing 100083, China Beijing Forestry University Beijing China

**Keywords:** *
Betula
*, Dermateaceae, new species, *
Prunus
*

## Abstract

*Dermea* was protected against its synonym, *Foveostroma*, due to its well-circumscribed generic concept and more frequent use. We describe and illustrate *Dermeachinensis***sp. nov.** based on its morphological characteristics and a molecular analysis of the internal transcribed spacer (ITS) and large subunit (LSU) sequence data. *Dermeachinensis* is isolated from *Betulaalbosinensis* with sexual and asexual morphs and can be distinguished from *D.molliuscula* on *Betula* trees by its aseptate and wider ascospores. The connection between the two morphs is proved based on sequence data. Here, we describe the asexual morph of *D.pruni* for the first time based on morphological and molecular data from the same host and country of origin, and compare it with other species of *Prunus*.

## Introduction

*Dermea* Fr. (Dermateaceae, Helotiales) was first proposed based on *D.cerasi* (Fries, 1825), which is the sexual Asexual morph of the type species of *Micropera* Lév. (Léveillé, 1846) and *Foveostroma* DiCosmo (DiCosmo 1978), namely *M.drupacearum* and *F.drupacearum*, respectively. Due to the well-circumscribed concept and its more frequent use, *Dermea* was protected as the legitimate generic name (Johnston et al. 2014).

Groves (1946) accepted 16 species in *Dermea* and proposed a key for this genus based mainly on the characteristics of apothecia, asci, ascospores, and conidia, along with host associations. Subsequently, *Dermeatumifaciens* (Ramakrishnan & Ramakrishnan, 1948), *D.pruni* (Groves, 1951), *D.grovesii* (Reid & Pirozynski, 1966), *D.rhytidiformans* (Funk & Kuijt, 1970), *D.tetrasperma* (Funk, 1976), *D.abietinum* (Johnston et al., 2014), *D.boycei* (Johnston et al., 2014), *D.stellata* (Johnston et al., 2014), and *D.persica* (Mehrabi et al., 2018) were added to this genus. However, *D.balsamea* and *D.peckiana*, which were accepted by Groves (1946), were later synonymised with *D.abietinum* and *D.stellata*, respectively (Johnston et al. 2014). Thus, 23 species were included in this genus before this study.

*Dermea* is a well-characterized genus with hard, leathery, dark brown to black apothecia; cylindrical to clavate-cylindrical, usually eight-spored asci; and ellipsoid-fusiform to ellipsoidal, hyaline to yellowish-brown, aseptate to 3-septate ascospores (Groves 1946; Mehrabi et al. 2018). The asexual Asexual morph of *Dermea* contains rather diverse conidiomatal structures, which usually accompany the apothecia (Groves 1946; Mehrabi et al. 2018). Additionally, two kinds of conidia are characterized: elongate-fusiform to sickle-shaped macroconidia and bacillary to filiform microconidia (Groves 1946; Mehrabi et al. 2018).

*Dermea* species are generally considered highly host-specific (Groves 1946, 1951). The plant genus *Prunus* is the major host for *Dermea*, with *D.cerasi*, *D.padi*, *D.prunastri*, and *D.pruni* described from them (Groves 1946, 1951). However, ascospores in *D.pruni* are larger than those from the other three species (Groves 1951). *Dermeacerasi*, *D.padi*, and *D.prunastri* can be easily distinguished by the macroconidial and microconidial dimensions (Groves 1946). Among these four species, *D.cerasi*, *D.padi*, and *D.prunastri* were recognized based on both sexual and asexual fruiting bodies (Groves 1946), but *D.pruni* was proposed only with a sexual Asexual morph based on a specimen (Teng #3352, preserved in the herbarium of the University of Michigan) collected from China (Groves 1951). Hence, the re-collection of *D.pruni* specimens aiming for an asexual Asexual morph from the original host and country seems meaningful. Additionally, few sequence data are available for most *Dermea* species, and considering that the host associations may be incorrect and that many geographical areas are still insufficiently studied, the synonymies and actual numbers of *Dermea* species are still unclear.

*Dermea* species were considered pathogenic to their hosts (Groves 1951; Abeln et al. 2000). For example, *D.abietinum* (syn. *D.balsamea*) caused hemlock dieback (Dodge 1932) and *D.prunastri* was considered the cause of greengage plums die-back (Dowson 1913). However, members of *Dermea* have not been recently reported to cause serious plant diseases.

During our fungal collection surveys conducted in China, we collected several *Dermea* specimens from two species of tree, *Betulaalbosinensis* and Prunuscerasiferaf.atropurpurea. We identified fungi species using both morphological and molecular approaches; as a result, a novel species and the asexual Asexual morph of *D.pruni* are described herein for the first time.

## Materials and methods

### Sample collections and fungal isolates

Fresh specimens of *Dermea* were collected from tree barks during our fungal collection trip in China. We obtained single ascospore and conidia isolates by removing a mucoid spore mass from apothecia or conidiomata and spreading the suspension on the surface of 2% malt extract agar (MEA; 20 g malt extract, 20 g agar, 1 L water). After inoculation, agar plates were incubated at 25 °C to induce germination of spores. Single germinating spores were then transferred to clean plates under a dissecting microscope with a sterile needle. Specimens and isolates were deposited in the Museum of Beijing Forestry University (BJFC). Axenic cultures are maintained in the China Forestry Culture Collection Center (CFCC).

### Morphological analysis

Species identification was based on the morphological characters of apothecia and conidiomata produced on natural substrates. Cross-sections were prepared manually using a double-edged blade under a Leica stereomicroscope (M205 FA). Photomicrographs were captured with a Nikon Eclipse 80i microscope equipped with a Nikon digital sight DS-Ri2 high-definition colour camera, using differential interference contrast (DIC) illumination and the Nikon software, NIS-Elements D Package 3.00. Measurements of ascospores and conidia are reported as the maximum and minimum in parentheses and the range representing the mean ± standard deviation of the number of measurements is given in parentheses. Cultural characteristics of isolates incubated on MEA in the dark at 25 °C were recorded.

### DNA extraction, PCR amplification and sequencing

Genomic DNA was extracted from axenic living cultures on MEA with cellophane using a modified CTAB method (Doyle and Doyle 1990). The internal transcribed spacer (ITS) region was amplified with primers ITS1 and ITS4 (White et al. 1990), and the large subunit (LSU) region with the primers LR0R and LR5 (Vilgalys and Hester 1990). Amplification of ITS and LSU were accomplished by an initial step of 2 min at 95 °C, followed by 35 cycles of 30 s at 95 °C, 30 s at 51 °C, and 40 s at 72 °C, with a final extension of 10 min at 72 °C. DNA sequencing was performed on an ABI PRISM 3730XL DNA Analyzer using BigDye Terminater Kit 3.1 (Invitrogen) at the Shanghai Invitrogen Biological Technology Company Limited (Beijing, China).

### Phylogenetic analyses

Sequences from this study and reference sequences obtained from GenBank (Table 1) were aligned and edited manually using MEGA6 (Tamura et al. 2013). The alignments were concatenated for phylogenetic analyses. Maximum parsimony (MP) analyses were conducted with PAUP 4.0b10 (Swofford 2003), using 1000 heuristic search replicates with random-additions of sequences along with the tree bisection and reconnection (TBR) branch swapping algorithm (MULTREES option in effect, steepest descent option not in effect). All molecular characters were unordered and given equal weight; analyses were performed with gaps treated as missing data; the COLLAPSE command was set to minbrlen, maxtrees were set to 5000. All equally parsimonious trees found were saved in the MP analyses. Other calculated parsimony scores were tree length (TL), consistency index (CI), retention index (RI), and rescaled consistency (RC). MP bootstrap analyses with 1000 replicates were performed in the same manner, with 10 rounds of heuristic search replicates with random addition of sequences and subsequent TBR branch swapping during each bootstrap replicate. ML analyses were conducted using RAxML (Stamatakis 2006) as implemented in raxmlGUI 1.3 (Silvestro and Michalak 2012), using the ML + rapid bootstrap setting and the GTRGAMMA substitution model with 1000 bootstrap replicates. Taxonomic novelties were deposited in MycoBank.

**Table 1.** Strains and NCBI GenBank accession numbers used in this study. Strains from this study are in bold.

Species	Strain	Genbank	
		ITS	LSU
* Davidhawksworthia ilicicola *	CBS 734.94	KU728517	KU728556
* Davidhawksworthia ilicicola *	CBS 261.95	KU728516	KU728555
* Dermea acerina *	CBS 161.38	AF141164	DQ247801
* Dermea ariae *	CBS 134.46	AF141158	NA
* Dermea cerasi *	CBS 136.46	AF141159	NA
*** Dermea chinensis ***	**CFCC 53008**	**MK330013**	**MK626645**
*** Dermea chinensis ***	**CFCC 53009**	**MK330014**	**MK626646**
*** Dermea chinensis ***	**CFCC 53010**	**MK330015**	**MK626647**
* Dermea hamamelidis *	CBS 137.46	AF141157	NA
* Dermea padi *	CBS 140.46	AF141160	NA
* Dermea persica *	MFLU 16-0259	MH104719	MH104720
* Dermea prunastri *	CBS 143.46	AF141162	NA
*** Dermea pruni ***	**CFCC 53006**	**MK330016**	**MK626648**
*** Dermea pruni ***	**CFCC 53007**	**MK330017**	**MK626649**
* Dermea viburni *	CBS 145.46	AF141163	NA
* Mollisia dextrinospora *	ICMP 18083	HM116746	HM116757
* Neofabraea inaequalis *	CBS 326.75	KR859081	KR858872
* Neofabraea kienholzii *	CBS 126461	KR859082	KR858873
* Neofabraea malicorticis *	CBS 122030	KR859086	KR858877
* Neofabraea perennans *	CBS 102869	KR859087	KR858878
* Pezicula aurantiaca *	CBS 201.46	KR859102	KR858893
* Pezicula cornina *	CBS 285.39	KR859163	KR858915
* Pezicula cinnamomea *	CBS 239.96	KR859124	KR858955
* Pezicula eucrita *	CBS 259.97	KR859179	KR858971
* Pezicula neosporulosa *	CBS 101.96	KR859223	KR859015
* Pezicula pseudocinnamomea *	CBS 101000	KR859235	KR859027
* Pezicula sporulosa *	CBS 224.96	KR859261	KR859053
* Phlyctema vincetoxici *	CBS 123727	KF251207	KF251710
* Phlyctema vincetoxici *	CBS 123743	KF251208	KF251711
* Pseudofabraea citricarpa *	CBS 130533	KR859281	KR859075
* Pseudofabraea citricarpa *	CBS 130297	KR859279	KR859073

## Results

### Phylogenetic analyses

**Figure 1.**
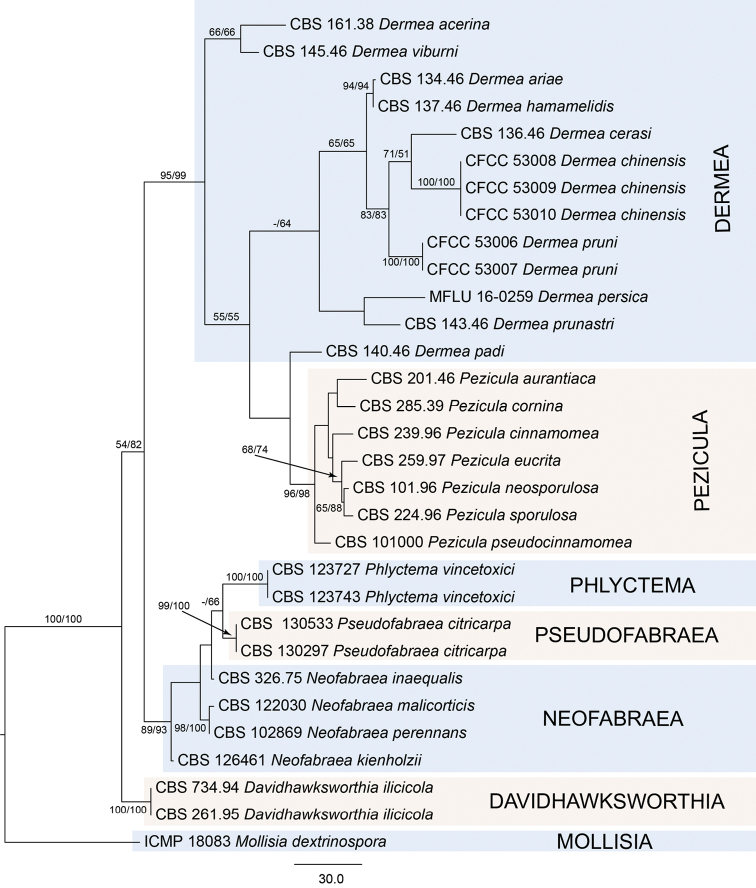
Phylogram of *Dermea* and related genera based on combined ITS and LSU sequence data. Values above or below the branches indicate maximum parsimony and maximum likelihood bootstrap support. Scale bar: 30 nucleotide substitutions.

The alignment based on the combined sequence dataset (ITS and LSU) contained 1431 characters. Of these, 1136 characters were constant, 103 variable characters were parsimony-uninformative, and 192 parsimony informative. The MP analyses resulted in five equally most parsimonious trees, with the first tree (TL = 601, CI = 0.647, RI = 0.807, RC = 0.522), which is shown in Figure 1. Tree topologies of the best tree revealed by the ML analyses was identical to those of the MP tree (not shown). The two species from this study appeared in two distinct clades, and three strains of *Dermeachinensis* from the *Betulaalbosinensis* cluster in a well-supported clade (MP/ML = 100/100) (Fig. 1).

### Taxonomy

#### Dermea chinensis

Taxon classificationAnimaliaHelotialesDermateaceae

C.M. Tian & N. Jiang sp. nov.

828880

Figures 2 , 3

##### Diagnosis.

*Dermeachinensis* differs from *D.molliuscula* by its wider ascospores

##### Holotype.

CHINA. SHAANXI PROVINCE, Ankang City, Huoditang forest park, 33°26'12"N, 108°26'42"E, 1650 m a.s.l., on branches of *Betulaalbosinensis*, N. Jiang & C.M. Tian leg., 18 Jul 2018 (holotype BJFC-S1729). Ex-type culture from sexual fruiting body: CFCC 53008; living culture from asexual fruiting body: CFCC 53009.

##### Etymology.

Named after the country where it was first discovered, China.

**Figure 2.**
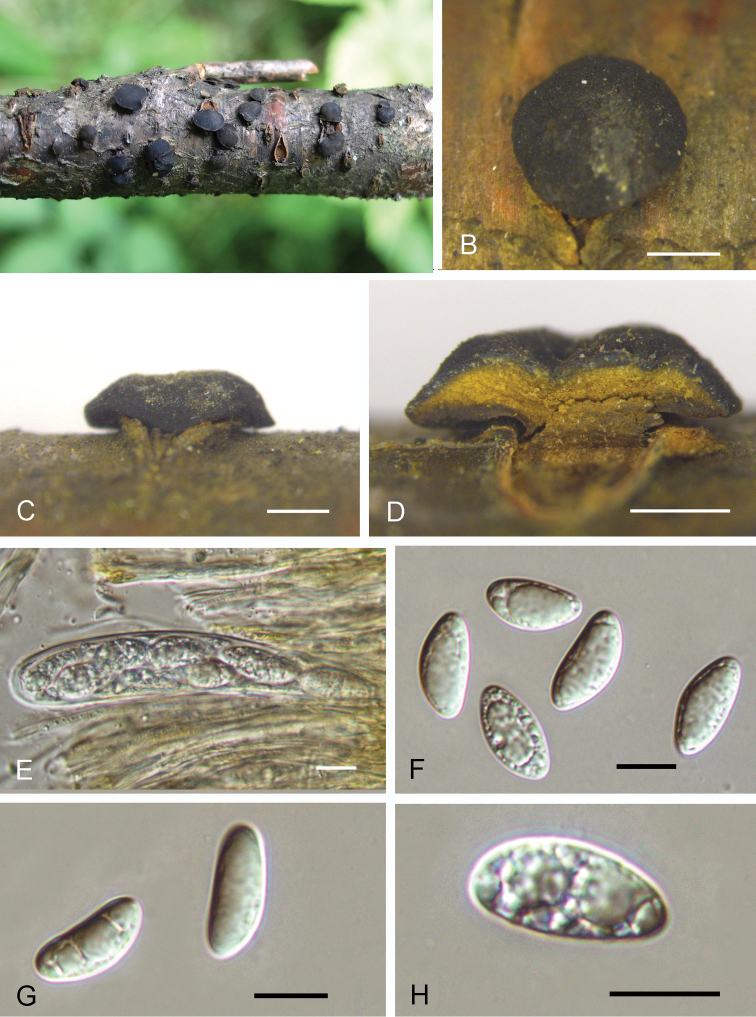
Sexual Asexual morph of *Dermeachinensis* from *Betulaalbosinensis* (BJFC-S1729, holotype) **A–C** apothecia on the natural substrate in surface view **D** longitudinal section through apothecium **E** ascus and paraphyses **F–H** ascospores. Scale bars: 1 mm (**B–D**); 10 μm (**E–H**).

**Figure 3.**
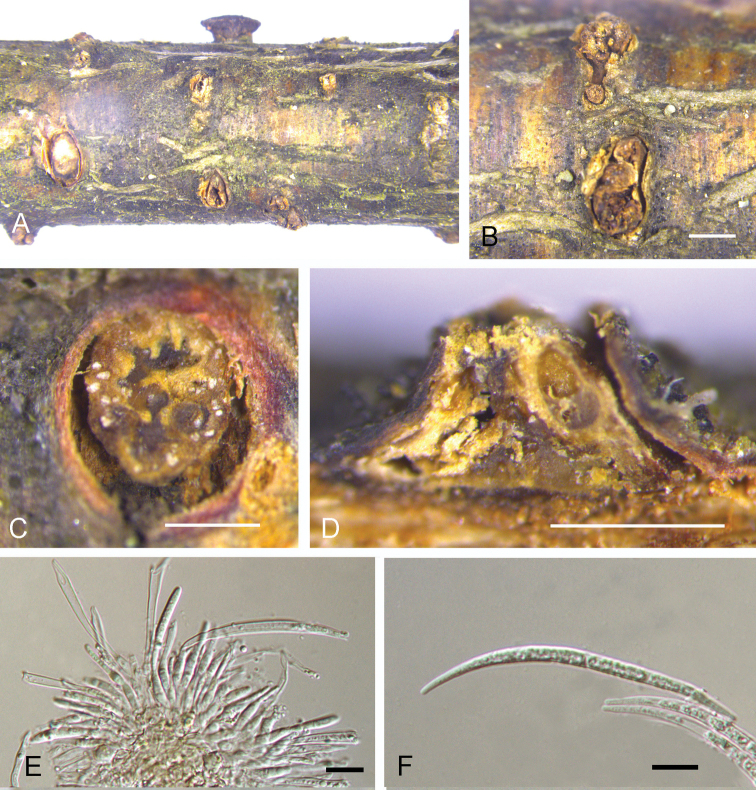
Asexual morph of *Dermeachinensis* from *Betulaalbosinensis* (BJFC-S1729, holotype) **A, B** conidiomata on the natural substrate in surface view **C** transverse section through conidioma **D** longitudinal section through conidioma **E, G** conidiophores **F, H** conidia. Scale bars: 1 mm (**B**); 0.5 mm (**C, D**); 10 μm (**E–H**).

##### Description.

*Sexual Asexual morph*: *apothecia* erumpent, scattered or sometimes gregarious, circular, sinuate, sessile to substipitate, 2.1–3.5 mm wide, 0.8–1.2 mm high (av. = 2.7 × 0.9 mm, *n* = 10), dark brown to black, hard, leathery to horny in consistency, hymenium at the first concave, becoming plane or convex, roughened, sometimes cracked, occasionally slightly umbilicate; tissue of the basal stroma pseudoparenchymatous, composed of closely interwoven hyphae with elongated cells about 8 μm in diameter, hyaline to brownish, thick walled, curving towards the outside, forming a darker, pseudoparenchymatous excipulum of thick-walled cells about 8 μm in diameter; subhymenium a narrow zone of closely interwoven hyphae about 3 μm in diameter. *Asci* 85–118 × 14–19 μm (av. x‒= 96.5 × 16.4 μm, *n* = 10), cylindric-clavate, tapering below into a short stalk, 8-spored. Paraphyses hyaline, filiform, septate, simple or branched, 1.5–2.5 in diameter, the tips slightly swollen up to 4 μm and glued together forming a yellowish epithecium. *Ascospores* (14.2–)16.3–17.1(–18.6) × (7.3–)7.5–8.5(–8.9) μm, l/w = (1.8–)1.9–2.2(–2.3) (*n* = 50), ellipsoid-fusiform, hyaline to yellowish-brown, straight or slightly curved, aseptate, irregular biseriate. *Asexual morph*: *conidial fruiting bodies* erumpent, gregarious, columnar to subconical, 0.5–2.5 mm wide, 0.4–0.7 mm high (av. = 1.6 × 0.6 mm, *n* = 10), yellowish, furfuraceous to glabrous, tearing open irregularly and widely at the top, waxy in consistency, more fresh when moist, usually containing 3–8 more or less lobed cavity. *Conidiophores* 7–18 × 2–3.5 μm, hyaline, aseptate, unbranched, tapering to a slender tip. *Conidiogenous cells* 5–15 × 1.5–3 μm, determinate, phialidic, cylindrical, hyaline. *Conidia* (54–)60–72(–78) × (3.2–)3.5–4(–4.2) μm, hyaline, fifiform, straight or curved, one-celled. *Microconidia* absent.

##### Culture characters.

On MEA at 25 °C colonies grow slowly, reaching 50 mm diameter within 60 d, pale yellow at first, gradually turning dark brown with scanty aerial mycelium.

##### Habitat and host range.

On dead corticated branches of *Betulaalbosinensis*.

##### Additional specimen examined.

CHINA. SHAANXI PROVINCE, Ankang City, Qinling Mountain, 33°26'12"N, 108°26'42"E, 1570 m a.s.l., on branches of *Betulaalbosinensis*, N. Jiang & C.M. Tian leg., 15 Jul 2018 (BJFC-S1730, living culture CFCC 53010).

##### Notes.

Three isolates of *D.chinensis* were obtained from *Betulaalbosinensis* cluster in a well-supported clade (MP/ML = 100/100) and appeared closely related to *D.cerasi* from *Prunus* branches. *Dermeachinensis* and *D.cerasi* are similar in macroconidia dimensions (54–78 × 3.2–4.2 μm in *D.chinensis* vs 40–60 × 2.5–4.5 μm in *D.cerasi*) but different in ascospore dimensions (14.2–18.6 × 7.3–8.9 μm in *D.chinensis* vs 15–20 × 5–7.5 μm in *D.cerasi*) and host associations (Groves 1946). Furthermore, the two species are separated by 51 bp differences in their ITS. *Dermeamolliuscula*, which occurs in the USA and Canada, is the other species inhabiting *Betula* trees. However, *D.chinensis* is distinguished from *D.molliuscula* by aseptate ascospores and in width (7.3–8.9 μm in *D.chinensis* vs 4–7 μm in *D.molliuscula*) (Groves 1946).

#### Dermea pruni

Taxon classificationAnimaliaHelotialesDermateaceae

(Teng) J.W. Groves, Mycologia 43(6): 721. 1952.

Figure 4

##### Description.

*Sexual Asexual morph*: see Groves (1952). *Asexual morph*: *conidial fruiting bodies* erumpent, gregarious, pulvinate, 0.6–2.3 mm wide, 0.2–0.35 mm high (av. = 1.8 × 0.28 mm, *n* = 10), yellowish, furfuraceous to glabrous, tearing open irregularly and widely at the top, waxy in consistency, more fresh when moist, usually containing up to 30 more or less lobed cavities. *Conidiophores* 4–15 × 1.5–2.5 μm, hyaline, aseptate, unbranched, tapering to a slender tip. *Conidiogenous cells* 3.5–15 × 1.5–2.5 μm, determinate, phialidic, cylindrical, hyaline. *Conidia* (62–)75–88(–95) × (2–)2.5–3.3(–3.5) μm, hyaline, fifiform, straight or curved, two-celled. *Microconidia* absent.

**Figure 4.**
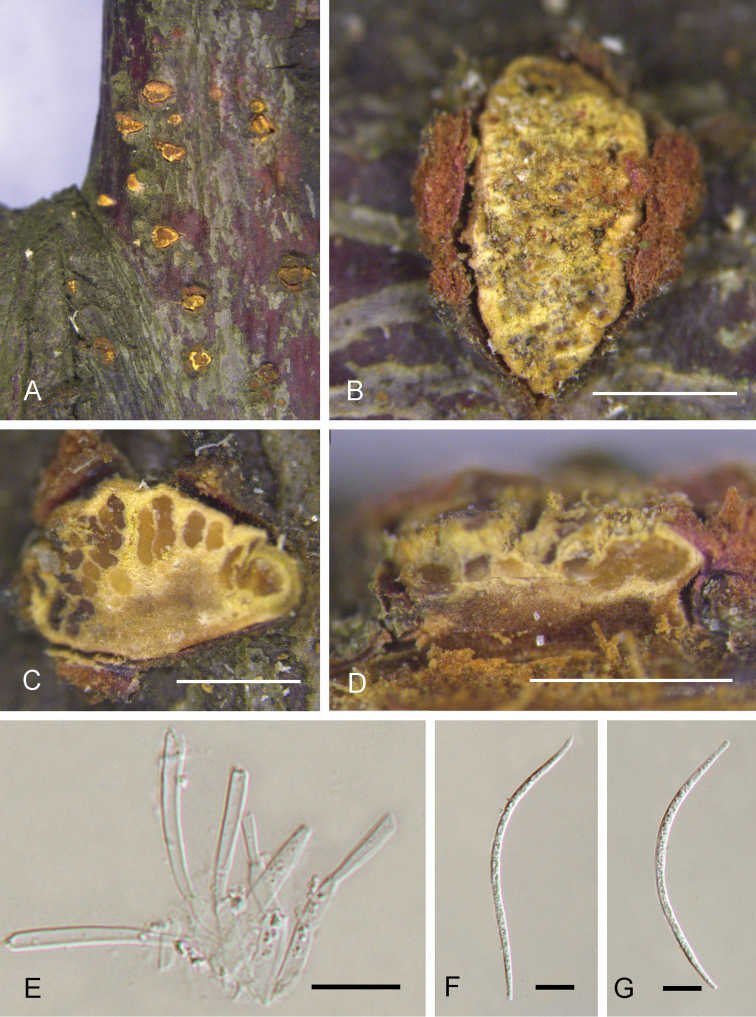
Asexual morph of *Dermeapruni* from Prunuscerasiferaf.atropurpurea (BJFC-S1727) **A, B** conidiomata on the natural substrate in surface view **C** transverse section through conidioma **D** longitudinal section through conidioma **E** conidiophores **F** conidia. Scale bars: 1 mm (**B, C**); 0.5 mm (**D**); 10 μm (**E, F**).

##### Culture characters.

On MEA at 25 °C colonies grow slowly, reaching 50 mm diameter within 50 d, at first pale yellow, gradually becoming dark brown with scanty aerial mycelium.

##### Habitat and host range.

On dying stems and branches of Prunuscerasiferaf.atropurpurea.

##### Specimens examined.

CHINA. SHAANXI PROVINCE, Ankang City, Qinling Mountain, 33°26'7"N, 108°26'48"E, 1570 m asl, on branches of Prunuscerasiferaf.atropurpurea, N. Jiang & C.M. Tian leg., 23 Jul 2018 (BJFC-S1727, living culture CFCC 53006). CHINA. SHAANXI PROVINCE, Ankang City, Qinling Mountain, 33°26'7"N, 108°26'48"E, 1570 m asl, on branches of Prunuscerasiferaf.atropurpurea, N. Jiang & C.M. Tian leg., 23 Jul 2018 (BJFC-S1728, living culture CFCC 53007).

##### Notes.

*Dermeapruni* was proposed based on a specimen collected from *Prunus* branches in Sichuan province, China. However, no living culture or DNA data were available (Groves 1951). In addition, the asexual Asexual morph was not included in the original description (Groves 1951). During our fungal collection trip in China, two *Dermea* specimens were accidentally discovered on a common road tree, Prunuscerasiferaf.atropurpurea in Shaanxi province, which borders Sichuan province, the original collection province of the holotype. Asexual fruiting bodies were observed on the whole trees, from stems to branches. However, no sexual asexual morph was found, even though we investigated all *Prunus* trees along the road. Conidial size was compared among our collections, *D.cerasi*, *D.padi*, and *D.prunastri*, which can distinguish them (Table 2). Considering that our collections and the type specimen (Teng #3352, preserved in the herbarium of the University of Michigan) of *D.pruni* were collected from the same hosts and from nearby regions (Groves 1951), our specimens were identified and treated here as *D.pruni*. However, more detailed taxonomic studies are needed, including DNA extraction from the holotype of *D.pruni* to compare ITS sequences of our collections and the holotype.

## Discussion

In this study, we collected several *Dermea* specimens from China and morphologically and molecularly examined them. *Dermeachinensis* from *Betula* trees is introduced, which can be distinguished from *D.molliuscula* by aseptate and wider ascospores, and from other species by host association (Table 2). Four *Dermea* species, *D.cerasi*, *D.padi*, *D.prunastri*, and *D.pruni* have been reported from *Prunus* trees (Groves 1946, 1951). These four species can be obviously distinguished by both morphological and molecular approaches. We update the asexual Asexual morph and molecular data of *D.pruni*.

**Table 2.** Comparison of phenotypic characters of currently accepted *Dermea* species.

Species	Host genera	Ascospores dimension (µm); septation	Macroconidia dimension (µm); septation	Microconidia dimension (µm)	Reference
* D. abietinum *	*Abies*; *Tsuga*	20–30 × 6–8; 1–4-celled	60–75 × 4–5; 1–4-celled	11–22 × 1.0–1.5	Groves 1946; Johnston 2014
* D. acerina *	* Acer *	13–20 × 5–8; 1–4-celled	15–25 × 5–8; 1-celled	6–10 × 1.0–2.0	Groves 1946
* D. ariae *	* Sorbus *	12–18 × 3–5; 1–4-celled	15–20 × 2.0–4.0; 1–2-celled	NA	Groves 1946
* D. bicolor *	* Amelanchier *	12–15 × 3–4; 1–2-celled	15–20 × 2.5–4.0; 1–2-celled	NA	Groves 1946
* D. boycei *	* Pseudotsuga *	16–28 × 4–7; 1–4-celled	42–56 × 3–4; 1–4-celled	8–14 × 1–2	Funk 1967; Johnston 2014
* D. cerasi *	* Prunus *	15–20 × 5–7.5; 1–4-celled	40–60 × 2.5–4.5; 1–2-celled	12–23 × 1.0–1.5	Groves 1946
* D. chinensis *	* Betula *	14–19 × 7–9; 1-celled	54–78 × 3.2–4.2; 1-celled	NA	This study
* D. chionanthi *	* Chionanthus *	18–25 × 7–9; 1–2(–4)- celled	25–35 × 5–7; 1–2-celled	NA	Groves 1946
* D. grovesii *	* Picea *	16.5–21.5 × 6–5; 1–3- celled	60–95 × 6.5–8; 7–11-celled	NA	Reid and Pirozynski 1966
* D. hamamelidis *	* Hamamelis *	15–20 × 5.0–7.5; 1–4- celled	18–25 × 4.5–6.0;1–2-celled	NA	Groves 1946
* D. libocedri *	* Libocedrus *	15–20 × 6–8; 1–4-celled	42–65 × 4–6; 1–4-celled	10–18 × 1.0–1.5	Groves 1946
* D. molliuscula *	* Betula *	15–20 × 4–7; 1–4-celled	50–75 × 2.5–3.5; 1–4-celled	7–12 × 1.0–1.5	Groves 1946
* D. padi *	* Prunus *	15–20 × 5–7; 1–4-celled	20–28 × 2.5–4.0; 1–2-celled	4–6 × 1.5	Groves 1946
* D. persica *	NA	NA	20–25 × 2.5–3.5; 1-celled	NA	Mehrabi et al. 2018
* D. piceina *	* Picea *	12–14 × 6–8; 1–2(–4)- celled	22–40 × 3–5; 1–4-celled	9–15 × 1.0–1.5	Groves 1946
* D. pinicola *	* Pinus *	13–18 × 5.0–7.5; 1–2- celled	30–40 × 4–6; 1–4-celled	NA	Groves 1946
* D. prunastri *	* Prunus *	15–20 × 5.0–7.5; 1–4- celled	20–30 × 5–7; 1-celled	7–10 × 1.5	Groves 1946
* D. pruni *	* Prunus *	15–20 × 8–10; 1(–4)-celled	62–95 × 2–3.5; 2-celled	NA	Groves 1951; This study
* D. rhytidiformans *	* Abies *	18–28 × 8–11; 1-celled	25–65 × 3.5–5.5; 1–4-celled	10–22 × 1.5	Funk and Kuijt 1970
* D. stellata *	* Nemopanthus *	12–18 × 4–6; 1–2(–4)- celled	40–55 × 2.5–4.5; 1–2-celled	8–13 × 1.5–2.0	Groves 1946; Johnston 2014
* D. tetrasperma *	* Pseudotsuga *	14–17 × 4–6; 1-celled	15–22 × 5–6; 1-celled	NA	Funk 1976
* D. tulasnei *	* Fraxinus *	15–20 × 6–8; 1–4-celled	25–40 × 6–8; 1-celled	NA	Groves 1946
* D. tumifaciens *	* Capparis *	13 × 5.4 / 10–19 × 4.8–9.6; 2-celled	18 × 7 / 15–22 × 4–9; 2- celled	NA	Ramakrishnan and Ramakrishnan 1948
* D. viburni *	* Viburnum *	14–18 × 3.5–5.5; 1–2- celled	30–45 × 2.5–4.0; 1–4-celled	NA	Groves 1946

The genus *Pezicula* is a phylogenetically close to *Dermea* species and has recently been confirmed based on an ITS-28S-16S rDNA analysis (Mehrabi et al. 2018). However, *Pezicula* is characterized by typically bright-coloured, yellowish to ochraceous, more fleshy-waxy apothecia, broader and more clavate asci, and more broadly ellipsoid to oblong-ellipsoid or ovoid ascospores (Grove 1946). Our phylogenenetic analysis of *Dermea* and related genera based on the combined ITS and LSU sequence data (Fig. 1) showed that *Pezicula* is well-supported as a separate clade with high values (MP/ML = 96/98). *Dermea* was thought to be a monophyletic group (Abeln et al. 2000), but *Dermea* was not well-supported, as *D.persica* was included in the analysis (Mehrabi et al. 2018). We added additional DNA sequence data in our study (Fig. 1), which indicates that *Dermea* is not monophyletic.

Species of *Dermea* are well-circumscribed by morphological characteristics. However, only 10 species (Table 1) are currently characterized by molecular data, and most species remain unconfirmed by phylogenetic examination. Hence, DNA data from type or ex-strains and newly obtained collections are essential in subsequent taxonomic work.

## Supplementary Material

XML Treatment for Dermea chinensis

XML Treatment for Dermea pruni
